# Surgical hip dislocation and sliding hip screw system for a femoral neck osteochondroma in multiple hereditary exostoses: a case report

**DOI:** 10.1186/s13256-026-06228-z

**Published:** 2026-06-18

**Authors:** Kevin Rama, Filippo Migliorini, Tommaso Bardazzi, Francesco Simeone, Michael Kurt Memminger

**Affiliations:** 1 Department of Orthopaedic and Trauma Surgery, Academic Hospital of Bolzano (SABES-ASDAA), 39100 Bolzano, Italy; 2 Department of Trauma and Reconstructive Surgery, University Hospital of Halle, Martin-Luther University Halle-Wittenberg, Ernst-Grube-Street 40, 06097 Halle (Saale), Germany; 3 Department of Life Sciences, Health, and Health Professions, Link Campus University, 00165 Rome, Italy; 4 Research Department of Clinical Orthopaedics, Eifelklinik St Brigida, Simmerath, Germany

**Keywords:** Multiple hereditary exostoses, Osteochondroma, Femoroacetabular impingement, Surgical hip dislocation, Sliding hip screw, Case report, Hip stability, Excision

## Abstract

**Background:**

Multiple Hereditary Exostoses (MHE) is a rare autosomal-dominant disorder characterized by the development of multiple osteochondromas. While often asymptomatic, these lesions can cause pain and functional limitations due to mechanical impingement or malignant transformation. Osteochondromas of the femoral neck pose unique challenges due to their proximity to critical neurovascular and weight-bearing structures.

**Case presentation:**

We present the case of a 43-year-old Italian woman with MHE who developed severe femoroacetabular impingement due to a large osteochondroma located at the inferomedial aspect of the left femoral neck. Surgical management included a safe surgical dislocation of the hip via trochanteric flip osteotomy, followed by complete resection of the lesion. Given the extensive cortical defect resulting from resection, a prophylactic sliding hip screw (SHS) system was implanted to prevent postoperative femoral neck fracture. Given the extensive resection and loss of the inferomedial femoral neck cortex, the postoperative recovery included initial non-weight bearing and a structured rehabilitation program. Histology confirmed a benign osteochondroma. At seven years postoperatively, the patient reported sustained pain relief, restored function, and preserved joint range of motion, with no radiographic or clinical complications.

**Conclusions:**

In cases of large femoral neck osteochondromas in MHE patients, surgical dislocation ensures safe and complete excision. When resection leads to substantial cortical weakening, prophylactic fixation with an SHS construct may offer mechanical stability, reduce fracture risk, and facilitate early mobilization. This combined approach may be particularly beneficial for joint preservation in young adults with structurally compromised femoral necks.

## Background

Multiple Hereditary Exostoses (MHE), also known as multiple hereditary osteochondromas, is an autosomal-dominant syndrome caused, in more than 90% of cases, by mutations in the EXT1 or EXT2 genes, resulting in multiple osteochondromas (exostoses) arising from the juxta-epiphyseal regions of long and flat bones [1]. Solitary osteochondromas represent the most common bone neoplasms in paediatric populations, accounting for approximately 10–15% of all primary bone tumours and 20–50% of benign bone lesions. In contrast, Multiple Hereditary Exostoses (MHE) is a rarer condition, with an estimated incidence ranging from 1:50,000 to 1:100,000. It exhibits near-complete penetrance, with most individuals developing multiple lesions during early adolescence [5]. Radiographically, osteochondromas are characterised by continuity between their cortex and medullary canal and the corresponding structures of the host bone, and are typically capped by hyaline cartilage. The diagnostic criteria for MHE include the presence of two or more osteochondromas in the patient or in a first-degree relative, although most affected individuals present with six or more lesions [5]. A major clinical concern in MHE is the potential for malignant transformation into secondary peripheral chondrosarcomas, with a lifetime risk of approximately 10%, significantly higher than the 1% risk associated with solitary lesions [2–8]. Osteochondroma dimensions and the width of its related cartilaginous cap are considered predictors of malignancy. Warning signs include new-onset pain after skeletal maturity, rapid lesion enlargement, irregular lesion margins, and cartilage cap thickness exceeding 2 cm in adults. Surveillance with radiography and cross-sectional imaging, especially MRI to measure cartilage cap thickness, is recommended when malignant change is suspected [3]. Exostoses are typically asymptomatic [2, 5, 6, 9]. However, they might cause chronic pain, impingement, and functional limitations if compression of the surrounding tissues occurs [2, 5, 6, 9].

## Case presentation

**Fig. 1:**
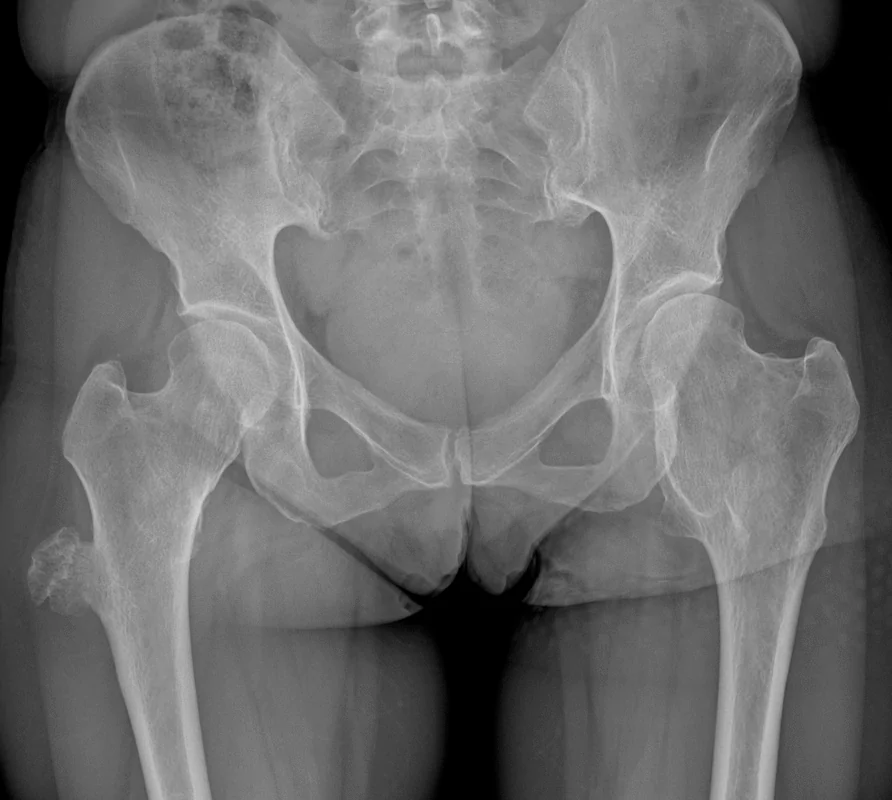
Anterior–posterior radiograph of the pelvis at admission. A large osteochondroma of the femoral neck at the calcar region causes the femoroacetabular impingement affecting the patient

A 43-year-old Italian woman affected by MHE syndrome was admitted to the Outpatient Clinic of the Department of Orthopaedic and Trauma Surgery at the Academic Hospital of Bolzano, Italy, complaining of increasing pain and severe functional limitations in the left hip. The patient was prescribed non-steroidal anti-inflammatory drugs (NSAIDs) daily for pain control. Anamnestic, the patient reported having undergone surgical resection of a secondary low-grade knee chondrosarcoma in the previous five years. During the clinical examination, posture and gait appeared physiologic, with no visible skin ailments or muscle hypotrophy. Palpation of key bony landmarks, such as the iliac crest, anterior superior iliac spine, greater trochanter, and ischial tuberosity, elicited no tenderness. The assessment of surrounding soft tissues revealed no tenderness or masses. There were no signs of hip infection. The range of motion of the hip was severely limited, particularly in flexion and internal rotation. The neurological examination was negative for sensorimotor deficits, and no evidence of peripheral vascular disease was found. A plain anteroposterior radiograph of the pelvis shows a femoroacetabular impingement caused by a large osteochondroma at the left femoral neck (Fig. 1).

**Fig. 2:**
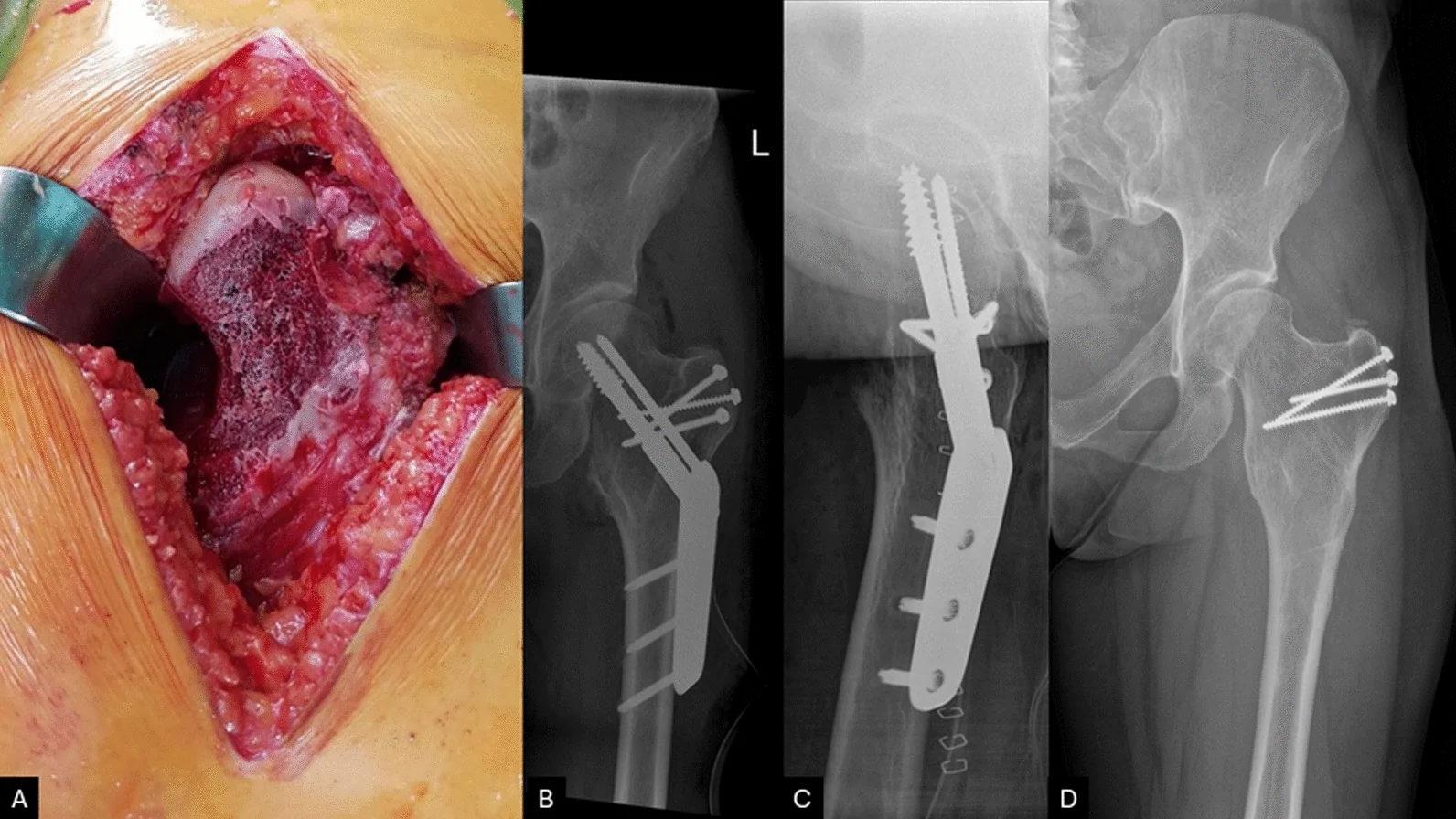
**a** Intra-operative image of the femoral neck following resection of the exostosis. The substance defect of the femoral neck is considerable, with almost half of the femoral neck being resected, including the cortex at the calcar region. This necessitated preventative fixation using a Dynamic Hip Screw. **b** Post-operative a.p. pelvis radiograph after resection of the exostosis via surgical hip dislocation and preventative fixation with an Anteversa sliding hip screw system. **c** Cross-table lateral post-operative radiograph highlighting the extent of the resection undertaken. **d** An anteroposterior radiograph of the pelvis, taken seven years after the initial surgery, reveals healing and remodelling of the femoral neck and trochanteric region; implant removal was carried out approximately 18 months after the initial procedure

The surgical intervention was performed by a single surgeon (MKM) with the help of assistant surgeons. With the patient under general anaesthesia and in the lateral decubitus position, a surgical hip dislocation with trochanteric osteotomy via a lateral approach was conducted in a previously published manner [10]. This approach provides 360-degree access to the femoral head and acetabulum while preserving the deep branch of the medial femoral circumflex artery to ensure adequate vascular supply. A straight lateral incision was then made over the greater trochanter. The approach involves a trochanteric flip osteotomy, in which a thin, wafer-like segment of the greater trochanter is osteotomised, maintaining the insertions of the gluteus medius and minimus at the trochanter and the vastus lateralis distally. This fragment is mobilised anteriorly without disrupting the external rotators or the posterior capsule. Following a Z-shaped capsulotomy, an anterior dislocation of the hip is performed. The osteochondroma of the femoral neck was resected first by conventional use of osteotomes. The remaining tumorous tissue was resected by using a 6 mm high-speed burr (Stryker Corp., Kalamazoo, MI, USA). Given the large bony defect left after resection (Fig. 2a) with the absence of the calcar cortex supporting the weight distribution from the torso to the leg, preventative fixation using a sliding hip screw system was performed. An 80 mm anteverted sliding hip screw (Anteversa Sliding Hip Screw System, Intrauma S.p.A., Rivoli, Italy) was used (Fig. 2b, c). The hip was then reduced, and the capsule repaired. The trochanteric osteotomy fragment is reattached using cortical screws, ensuring stable fixation. The resected bone was sent to the Pathology Department of the Academic Hospital of Bolzano, Italy, for histological analysis.

Given the extensive resection and loss of the inferomedial femoral neck cortex, the postoperative recovery included an initial non-weight-bearing status for approximately the first eight weeks, and free range of motion was allowed, as tolerated by the patient based on the pain level. Physiotherapy was initiated after approximately three weeks, to help regain normal hip function, independence and quality of life. After the appearance of beginning consolidation of the inferomedial cortex at the femoral neck, partial weight-bearing, followed by full weight-bearing, was permitted, and strengthening of the hip abductors and core stability was advised. Indomethacin 100 mg daily was administered for ten days as a prophylaxis against heterotopic ossification, and Enoxaparin 4000 IU was given as prophylaxis for thromboembolism, in accordance with standard clinical practice and medico-legal guidelines commonly followed in Italy. The histological analysis confirmed the resected exostosis to be a benign osteochondroma. In particular, the report noted hyperplastic bone without signs of atypical cell morphology and no evidence for malignant transformation. Approximately 18 months postoperatively, the patient’s quality of life significantly improved, with no symptoms of pain or impingement. The range of motion included flexion at 90°, internal rotation at 5°, and external rotation at 45°; adduction and abduction were not limited. The patient underwent hardware removal 24 months postoperatively. These findings were confirmed at the seven-year follow-up. The range of motion (ROM) remains similar. She has noted increasing pain during daily activities in recent years; however, it is still considerably less than before the index surgery. We attribute the increased pain during physical activity to an irritation of the fascia latae at the greater trochanter on the left side, as the patient reported marked tenderness upon pressure on the region [11]. At the inguinal level, the patient reported no pain, and the hip ROM has not diminished in a clinically relevant manner, which is what prompts us not to attribute this pain to the hip articulation. Also, when comparing the latest radiography available, at seven years post-operatively (Fig. 2d), there is not a particular difference on the volume of the inferomedial femoral neck when comparing it to the post-operative radiography (Fig. 2b). A simple local injection with steroid and lidocaine improved the symptoms, so we attribute this pain to a greater trochanteric pain syndrome, perhaps due to the remaining trochanteric screws following the surgical hip dislocation.

## Discussion and conclusions

Surgical dislocation using the trochanteric flip osteotomy has become the standard approach for gaining full access to proximal femoral osteochondromas, particularly in hereditary multiple exostoses (MHE). Sorel et al. reported treatment of 20 femoral neck osteochondromas via Ganz dislocation in 17 MHE patients, observing significant improvements in hip range of motion and pain at a mean follow-up of 46 months. However, complications such as trochanteric nonunion, fractures, or avascular necrosis occurred [12]. Similarly, Shin et al. described a series including nine paediatric MHE hips treated with osteochondroplasty and dislocation, showing improved flexion from a mean of 83° to 109°, with no long-term avascular necrosis events [13]. Osteochondroma resection via dislocation allows for maximal visualization and safe excision, though extensive cortical removal may structurally weaken the femoral neck. Although no studies have specifically evaluated sliding hip screw (e.g., Dynamic Hip Screw, DHS by Synthes GmbH, Oberdorf, Switzerland) reinforcement in MHE dislocations, biomechanical and clinical literature on femoral neck fracture prophylaxis supports the use of DHS following corticectomy. For example, FAITH and related studies consistently favour DHS constructs to protect against mechanical failure under axial and rotational loads [14]. To date, no cohort or comparative studies have explicitly examined combining surgical dislocation with prophylactic DHS stabilization after osteochondroma removal in MHE. This gap justifies our case report, which integrates a traditional surgical dislocation approach with sliding hip screw reinforcement to maintain structural integrity in the context of extensive cortical resection. Regarding the surgical options, a small osteochondroma causing hip impingement could be resected through hip arthroscopy [15, 16]. However, exostoses of the femoral neck, especially if large, could create a mechanical impingement and necessitate open hip dislocation [17]. The osteochondroma was located at the inferomedial aspect of the femoral neck, a region critical for transmitting compressive forces across the hip joint during weight-bearing. This anatomical consideration highlights the mechanical vulnerability resulting from extensive cortical resection in that area. It justifies the use of prophylactic support with a sliding hip screw system to maintain structural integrity and prevent postoperative fracture. The biomechanical literature on this topic is limited; one biomechanical study on preventative fixation of the femoral neck in osteoporotic patients comparing different osteosynthesis devices provides additional information [18], although the authors finally concluded that more studies regarding this topic are needed. The sliding hip screw system is commonly used in orthopaedic and trauma surgery, is relatively easy to implant, and represents an effective device to gain stability in such situation [19, 20]. In addition, augmenting the resection using the sliding hip screw system allows early weight-bearing, which fasten post-operative recovery and improves patient satisfaction. In older adults, a complete resection of the femoral neck followed by a total hip replacement could be considered, though hip preservation surgery should be preferred when facing such patients [21]. Surgical dislocation and a sliding hip screw system in selected patients for femoroacetabular impingement caused by an osteochondroma of the femoral neck, within the context of multiple hereditary exostoses, may be effective in restoring quality of life and facilitating participation in daily activities.

## Data Availability

No data were generated during this study.

## Abbreviations

DHS: Dynamic hip screw
MHE: Multiple hereditary exostoses
NSAID: Non-steroidal anti-inflammatory drug(s)
ROM: Range of motion
SHS: Sliding hip screw
VTE: Venous thromboembolism
